# Analysis of an Indian diabetes prevention programme on association of adipokines and a hepatokine with incident diabetes

**DOI:** 10.1038/s41598-021-99784-x

**Published:** 2021-10-13

**Authors:** Priscilla Susairaj, Chamukuttan Snehalatha, Arun Nanditha, Krishnamoorthy Satheesh, Arun Raghavan, Ramachandran Vinitha, Ambady Ramachandran

**Affiliations:** India Diabetes Research Foundation, Dr. A. Ramachandran’s Diabetes Hospitals, 110, Anna Salai, Guindy, Chennai, 600 032 India

**Keywords:** Biochemistry, Immunology, Biomarkers, Medical research

## Abstract

To study the association and possible predictive role of visfatin, resistin, fetuin-A and chemerin with incident type 2 diabetes (T2DM) among Asian Indians with prediabetes. Their association with insulin resistance, β-cell function, glycaemia and anthropometry were also studied. This is a nested case–control study of a large 2-year prospective prevention trial in persons at high risk of developing T2DM. Baseline HbA1c values between 6.0% (42 mmol/mol) and 6.2% (44 mmol/mol) were chosen for this analysis (n = 144). At follow-up, persons with incident T2DM (HbA1c ≥ 6.5%, 48 mmol/mol) were grouped as cases (n = 72) and those reverted to normoglycaemia, (HbA1c < 5.7% (39 mmol/mol) as controls (n = 72). Insulin resistance showed the strongest association with incident T2DM ((Odds Ratio (OR): 23.22 [95%CI 6.36–84.77]; *p* < 0.0001). Baseline visfatin (OR: 6.56 [95%CI 2.21–19.5]; *p* < 0.001) and fetuin-A (OR: 1.01 [95%CI (1.01–1.04)]; *p* < 0.0001) independently contributed to the conversion of prediabetes to T2DM. The contribution was significantly higher when their elevated levels coexisted (OR: 12.63 [95%CI 3.57–44.63]; *p* < 0.0001). The area under the curve was 0.77 ± SE 0.4 (95%CI 0.69–0.85) and 0.80 ± SE 0.04 (95%CI 0.73–0.88) for visfatin (median 17.7 ng/ml, sensitivity and specificity: 75%, *p* < 0.0001) and fetuin-A (mean 236.2 µg/ml, sensitivity: 71%, specificity: 75%, *p* < 0.0001) respectively. Higher baseline visfatin and fetuin-A concentrations are strongly associated with incident T2DM and are predictive of future diabetes.

## Introduction

There have been extensive studies on clinical markers for type 2 diabetes (T2DM) involving glucose measurements, anthropometry, β-cell derived proteins and surrogate indices of insulin action and inflammation. These markers predict early metabolic abnormalities and disease progression with moderate sensitivity and specificity^1–6^.

Prior to the actual onset of dysglycaemia, altered levels of specific intermediary bioactive molecules influence insulin sensitivity thereby altering its action on the target tissues. Identifying such parameters could be of clinical significance in predicting future risk of T2DM.

We have shown that high circulating level of adiponectin improve insulin sensitivity and is an established protective factor of T2DM^7,8^, while elevated levels of few other adipocytokines namely interleukin-6 and retinol binding protein-4 alter normal glucose homeostasis^7,9^.

Previous studies showed that no single factor can precisely assess the individuals’ risk status of diabetes^4,7,10^. It is important to correlate circulating levels of cytokines with clinical markers of glucose and lipid metabolism, markers of insulin action and anthropometric profile to present a lucid representation of an individual’s risk profile. Prospective studies are useful to identify markers that might serve as potential targets for detecting future risk of T2DM and related co-morbid conditions.

We used web sources and data informatics tools to find peptides associated with the development of T2DM. From a panel of search results, we chose visfatin and resistin for their roles in glucose homeostasis^11,12^. These adipokines are correlated with markers of obesity, metabolic syndrome and cardiovascular diseases^13–15^. Fetuin-A, a hepatokine attenuates insulin signaling in the target tissues. It also combines with free fatty acids (FFAs) inducing apoptic signals in the β-cells of the pancreas reducing insulin secretion^16,17^. Chemerin is known to regulate insulin resistance, glucose and lipid metabolism and is a surrogate marker for hypertiglyceridaemia in T2DM^18,19^.

The primary objective of this analysis was to study the association of visfatin, resistin, fetuin-A and chemerin with incident T2DM. The secondary objectives were (1) to study the association of these parameters with insulin resistance and β-cell function, (2) correlate their levels with baseline measurements of fasting plasma glucose (FPG), glycated haemoglobin (HbA1c), plasma insulin, body fat composition and waist circumference (WC) and (3) to evaluate their predictive roles for T2DM.

## Results

A total of 144 participants (men: 115, women: 29) with prediabetes, specifically with HbA1c values between 6.0% (42 mmol/mol) and 6.2% (44 mmol/mol) at baseline were selected for this analysis. This was to avoid the confounding effect of higher HbA1c values on the conversion rate to diabetes. The details of sample selection, clinical and laboratory analyses are shown under material and methods. During follow-up, persons converted to T2DM (HbA1c ≥ 6.5%, 48 mmol/mol) were grouped as cases (n = 72) and those reverted to normoglycaemia, (HbA1c < 5.7% (39 mmol/mol) were grouped as controls (n = 72). The characteristics of the study cohort at the baseline and at the endpoint of the study are shown in Table 1. Mean age of the total study cohort was 45.6 ± 4.9 years. The body mass index (BMI) (27.5 ± 3.3 kg/m^2^) and WC (men: 95.5 ± 7.4 cms; women: 93.8 ± 8.1 cms) were increased at baseline due to the selection criteria of the participants in primary study.

**Table 1:** General characteristics of the study groups—baseline and follow up.

Variables	Total prediabetes at baseline	Reverted to normoglycaemia			Converted to diabetes		
		Baseline	Follow up	*P* value	Baseline	Follow up	*P* value
n	144	n = 72			n = 72		
Male:female	115:29	61 :11			54:18		
Age (years)	45.6 ± 4.9	45.2 ± 4.4			46.1 ± 5.3		
Body Mass Index (kg/m^2^)	27.5 ± 3.3	26.5 ± 2.3	26.2 ± 2.6	0.01	28.5 ± 3.8^**‡**^	28.8 ± 3.9^**‡**^	0.01
**Waist circumference (cm)**							
Male	95.5 ± 7.4	93.6 ± 6.4	92.9 ± 6.7	0.02	97.6 ± 8.1^**†**^	97.8 ± 8.1^**†**^	0.53
Female	93.8 ± 8.1	89.5 ± 5.1	89.4 ± 3.8	0.88	96.4 ± 8.5^**†**^	97.6 ± 8.6^**†**^	0.05
**Body fat (%)**							
Male	28.6 ± 3.7	27.9 ± 3.5	27.4 ± 3.6	0.21	29.4 ± 3.7^**†**^	29.7 ± 3.9^**†**^	0.22
Female	38.6 ± 3.8	36.7 ± 3.5	36.8 ± 3.5	0.85	39.7 ± 3.6^**†**^	40.0 ± 3.4^**†**^	0.31
Visceral fat (%)	13.4 ± 4.4	12.3 ± 3.3	12.3 ± 3.7	0.64	14.5 ± 5.6^**†**^	15.7 ± 5.9^**‡**^	0.001
Subcutaneous fat (%)	22.9 ± 6.7	21.2 ± 5.1	20.9 ± 5.3	0.17	21.5 ± 7.7^**†**^	24.9 ± 7.8^**‡**^	0.27
Skeletal muscle (%)	27.6 ± 3.3	28.3 ± 2.9	28.3 ± 2.9	0.77	27.0 ± 3.5^**†**^	26.8 ± 3.5^**†**^	0.18
Fasting plasma glucose (mg/dl)	96.8 ± 13.6	91.0 ± 11.4	93.4 ± 9.4	0.14	102.6 ± 13.5^**‡**^	112.3 ± 18.6^**†**^	< 0.0001
HbA1c (%)	6.1 ± 0.1	6.1 ± 0.1	5.5 ± 0.1	< 0.0001	6.1 ± 0.1	7.0 ± 0.4^**‡**^	< 0.0001
**Lipid profile (mg/dl)**							
Triglycerides*	123.0 (86.3–165.5)	120.0 (89–157.8)	117.5(90.3–165)	0.84	123.5(85.3–170)	140.5 (103.2–199.5)	0.001
Cholesterol	178.03 ± 32.7	180.0 ± 33.5	181.6 ± 33.6	0.66	179.5 ± 31.9	192.0 ± 35.6	0.01
LDL-Chol	111.16 ± 28.3	111.9 ± 27.1	111.8 ± 28.2	0.98	110.4 ± 29.5	111.5 ± 30.7	0.64
HDL-Chol	39.0 ± 7.8	39.5 ± 8.5	42.4 ± 10.2	< 0.0001	38.6 ± 7.0	40.6 ± 9.4	0.05
Visfatin (ng/ml)*	17.73 (12.7–25.1)	14.18 (12.8–17.8)	16.6 (15.2–18.8)	< 0.0001	22.7(16.9–28.4)^**‡**^	24.4 (16.3–28.3)^**‡**^	0.48
Resistin (ng/ml)*	7.1 (5.3–9.5)	6.7 (5.3–8.6)	5.8 (3.9–7.5)	< 0.0001	7.2 (5.1–9.4)	8.8 (6.2–12.5)^**‡**^	< 0.0001
Fetuin-A (µg/ml)	236.16 ± 55.8	208.9 ± 38.9	199.6 ± 19.6	0.23	263.5 ± 57.1^**‡**^	276.2 ± 54.8^**‡**^	0.004
Chemerin (ng/ml)	127.58 ± 37.7	125.3 ± 40.1	120.2 ± 42.1	0.10	129.8 ± 35.4	151.7 ± 43.7	< 0.0001
HOMA-IR*	2.9 (1.8–4.5)	2.4 (1.6–3.6)	2.4 (1.7–3.3)	0.77	4.2 (2.5–5.8)^**‡**^	3.9 (2.8–5.4)^**‡**^	0.89
HOMA β*	145.5 (89.6–217.3)	144 (85.4–220.7)	120.1 (92.6–169.2)	0.12	163.5 (98.3 –212.6)	105.3 (72.8–165.6)	0.002

*Median (IQR) for non-normally distributed variables/Mean ± SD for normally distributed variables.

Intra-group comparison (Baseline vs. Follow up) - Paired t test (parametric), Wilcoxon signed-rank Test (non-parametric).

Inter-group comparison (NGT vs. DM) - Un-Paired t test (parametric): ^†^*p* value < 0.05, ^‡^*p* value < 0.0001, Mann–Whitney (non-parametric): ^†^*p* value < 0.05, ^‡^*p* value < 0.0001.

*HbA1c* glycosylated haemoglobin, *LDL-Chol* low density lipid cholesterol, *HDL-Chol* high density lipid cholesterol, *HOMA-IR* homeostatic model assessment of insulin resistance, *HOMA* *β* homeostatic model assessment of β-cell function.

Baseline and follow-up values were higher among persons who converted to T2DM when compared with the normoglycaemia group. Participants with T2DM had significantly higher body fat, visceral and subcutaneous fat but lower skeletal muscle mass at baseline and at follow-up when compared to normoglycaemic persons. The baseline HbA1c was similar in the two groups (6.1% ± 0.1), at follow-up, the mean values were 5.5 ± 0.1% (37 mmol/mol) and 7.0 ± 0.4% (53 mmol/mol) in the normoglycaemic and T2DM groups respectively. The T2DM group had significantly higher FPG at baseline (102.6 ± 13.5 mg/dl) compared to the normoglycaemic group (91.0 ± 11.4, *p* < 0.0001). Lipid parameters were similar at baseline in both groups, at follow-up, a significant increase in serum triglyceride and cholesterol levels were seen in the T2DM. High density lipoprotein cholesterol (HDLc) improved in both groups at follow-up. Persons with T2DM showed higher insulin resistance than the normoglycaemic group at both baseline (*p* < 0.0001) and at follow-up (*p* < 0.0001). At baseline, the β-cell function was similar in the two groups and a significant decline was seen among T2DM at follow-up (*p* < 0.05).

The median concentration of visfatin at baseline was 17.7 (12.7–25.1 ng/ml) in the total study group. Higher levels of visfatin were observed among the T2DM group at both baseline (*p* < 0.0001) and at follow-up (*p* < 0.0001) when compared to persons reverted to normoglycaemia.

The latter group showed an increase in visfatin levels at follow-up compared to its baseline concentrations (*p* < 0.0001). The median level of resistin in the total group was 7.1 (5.3–9.5) ng/ml at baseline. During follow-up, its level decreased significantly among those reverted to normoglycaemia (*p* < 0.0001), whereas it increased among the T2DM group (*p* < 0.0001). At baseline, the mean concentration of fetuin-A in the total group was 236.2 ± 55.8 µg/ml. Persons with T2DM had higher concentrations at baseline (*p* < 0.0001) and at follow-up (*p* < 0.0001) when compared to the normoglycaemic group. Its circulating levels further increased during follow-up in the T2DM group (*p* = 0.004). The mean concentration of chemerin in the total group at baseline was 127.6 ± 37.7 ng/ml. The two groups showed similar concentrations at baseline and at follow-up. In comparison to its baseline value, chemerin concentrations were significantly higher at follow-up in the T2DM group (*p* < 0.0001). During the course of the study, no significant differences in the concentrations of fetuin-A and chemerin were observed in persons reverted to normoglycaemia.

Table 2 shows the correlations of visfatin, resistin, fetuin-A and chemerin with other risk factors studied at baseline. Serum resistin and chemerin were positively correlated with components of body fat; total body fat, visceral fat and subcutaneous fat mass, while serum visfatin was correlated with visceral fat mass alone. Skeletal muscle mass showed an inverse association with resistin and chemerin. Measures of insulin resistance; fasting plasma insulin and homeostasis model of assessment for insulin resistance (HOMA-IR) were positively correlated with serum visfatin, resistin and fetuin-A. The index of β-cell function, homeostasis model of assessment for β-cell function (HOMA-β) was inversely correlated with serum resistin while with visfatin it was positively correlated. Among the study variables serum visfatin and fetuin-A were mutually correlated.

**Table 2:** Correlation of Visfatin, Resistin, Fetuin-A and Chemerin with other variables studied.

Variables	Visfatin		Resistin		Fetuin-A		Chemerin	
	r	*P* value	r	*P* value	r	*P* value	r	*P* value
**Serum peptides**								
Visfatin (ng/ml)*	–	–	0.15	0.08	0.19	**0.02**	0.16	0.06
Resistin (ng/ml)*	0.15	0.08	–	–	0.10	0.23	0.03	0.73
Fetuin–A (µg/ml)	0.19	**0.02**	0.10	0.23	–	–	0.08	0.34
Chemerin (ng/ml)	0.16	0.06	0.03	0.73	0.08	0.34	–	–
**Anthropometric variables**								
Body Mass Index (kg/m^2^)	0.31	< **0.0001**	0.48	< **0.0001**	0.14	0.10	0.23	**0.007**
Waist Circumference (cm)	0.32	< **0.0001**	0.46	< **0.0001**	0.18	**0.03**	0.13	0.12
Body Fat (%)	0.10	0.23	0.32	< **0.0001**	0.13	0.11	0.18	**0.03**
Visceral Fat (%)	0.33	**< **0.0001	0.42	< **0.0001**	0.15	0.07	0.17	**0.04**
Subcutaneous Fat (%)	0.08	0.35	0.30	< **0.0001**	0.12	0.17	0.18	**0.03**
Skeletal muscle (%)	− 0.01	0.96	− 0.23	**0.006**	− 0.11	0.20	− 0.16	0.05
**Biochemical parameters**								
Fasting Plasma Glucose (mg/dl)	0.10	0.23	− 0.02	0.86	0.27	**0.001**	− 0.01	0.89
HbA1c (%)	0.16	0.05	0.03	0.70	0.14	0.10	0.02	0.86
Triglycerides	− 0.05	0.54	0.03	0.72	0.13	0.13	− 0.08	0.36
Cholesterol	− 0.10	0.24	− 0.04	0.64	0.12	0.17	− 0.04	0.64
LDL-Chol	− 0.11	0.19	− 0.08	0.36	0.09	0.27	− 0.03	0.74
HDL-Chol	0.02	0.78	0.04	0.67	− 0.09	0.28	0.08	0.33
Fasting plasma insulin (mU/L)*	0.26	**0.002**	0.20	**0.01**	0.29	< **0.0001**	0.04	0.68
HOMA-IR*	0.26	**0.001**	0.19	**0.02**	0.34	< **0.0001**	0.03	0.73
HOMA—β*	0.18	**0.03**	− 0.11	0.19	0.09	0.28	0.06	0.49

*Median values are log transformed. Values shown are correlation coefficients (r).

Correlation is considered significant at *p* < 0.05 and the respective values are in bold.

*HbA1c* glycosylated haemoglobin, *LDL-Chol* low density lipid cholesterol, *HDL-Chol* high density lipid cholesterol, *HOMA-IR* homeostatic model assessment of insulin resistance, *HOMA* *β* homeostatic model assessment of β-cell function.

Multiple logistic regression analyses were performed to study the variables associated with incident T2DM. The variables that correlated with visfatin, resistin, fetuin-A and chemerin in the univariate analyses were included. The variables that were significantly associated with the progression to T2DM are shown in Table 3. As expected, insulin resistance showed the strongest association with incident diabetes ((Odds Ratio (OR): 23.22 [95%CI 6.36–84.77]; *p* < 0.0001).

**Table 3:** The associations of study parameters and other risk factors with incident diabetes—results of multiple logistic regression analyses.

Variables*	β	Odds ratio	95% Confidence Interval		*p* value
			Lower	Upper	
Visfatin (ng/ml)	1.88 (0.56)	6.56	2.21	19.5	0.001
Fetuin-A (µg/ml)	0.03 (0.01)	1.01	1.01	1.04	< 0.0001
Visfatin ng/ml.FetuinA µg/ml	2.54 (0.64)	12.63	3.57	44.63	< 0.0001
Body Mass Index (kg/m^2^)	0.68 (0.20)	2.0	1.33	2.93	0.001
Visceral Fat (%)	− 0.46 (0.17)	0.63	0.43	0.88	0.006
HOMA-IR	3.15 (0.66)	23.22	6.36	84.77	< 0.0001
HOMA-β	− 2.37 (0.57)	0.09	0.03	0.29	< 0.0001

Dependent variable: T2DM versus Normal.

*Variables that were significantly associated with incident diabetes are shown in the table.

Baseline levels of visfatin (OR: 6.56 [95%CI 2.21–19.5]; *p* < 0.001) and fetuin-A (OR: 1.01 [95%CI (1.01–1.04)]; *p* < 0.0001) independently contributed to the conversion of prediabetes to T2DM. The contribution to incident T2DM was significantly higher when elevated baseline levels of visfatin and fetuin-A coexisted (OR: 12.63 [95%CI 3.57–44.63]; *p* < 0.0001). Inverse associations were observed with HOMA-β (OR: 0.09 [95%CI 0.03–0.29]; *p* < 0.0001) and visceral fat mass (OR: 0.63 [95%CI 0.43–0.88]; *p* = 0.006). BMI was the only anthropometric factor that was associated with the conversion to T2DM (OR: 2.0 [95%CI 1.33–2.93]; *p* < 0.001). Circulating concentrations of serum resistin and chemerin, lipids, FPG, WC and body fat components except for visceral fat mass did not show an association with incident T2DM.

**Figure 1:**
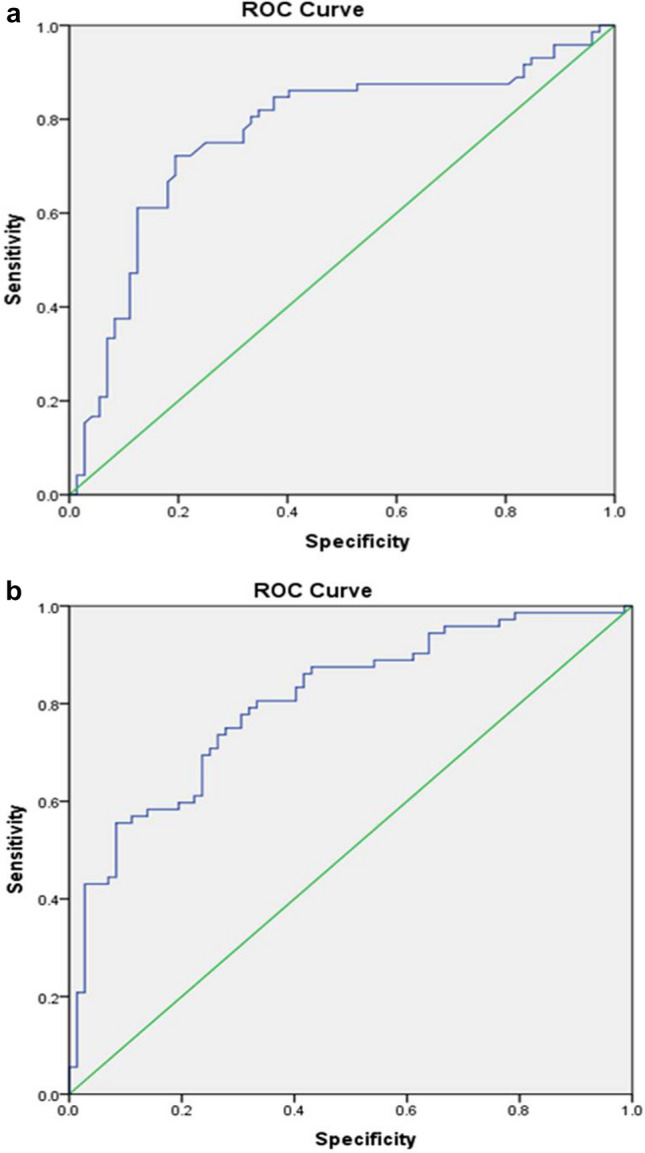
(**a**) Receiver Operating Curve showing predictive role of baseline Visfatin for incident diabetes. The median value of Visfatin was 17.7 ng/ml (sensitivity 75%, specificity 75% *p* < 0.0001). AUC = 0.77 ± SE 0.4 (95%CI 0.69–0.85). (**b**) Receiver Operating Curve showing predictive role of baseline Fetuin-A for incident diabetes. The mean value of Fetuin-A was 235.5 µg/ml (sensitivity 71%, specificity 75% p =  < 0.0001). AUC = 0.80 ± SE 0.04 (95%CI 0.73–0.88).

The predictive roles of visfatin and fetuin-A for incident T2DM were also studied using the receiver operating curve (ROC) analysis. For a median value of 17.7 ng/ml, visfatin had sensitivity and specificity of 75%, *p* < 0.0001. The area under the curve (AUC) was 0.77 ± SE 0.4 (95% CI 0.69–0.85) (Fig. 1a). For a mean value of 236.2 µg/ml, fetuin-A showed a sensitivity of 71% and a specificity of 75%, *p* < 0.0001. The AUC was 0.80 ± SE 0.04 (95% CI 0.73–0.88) (Fig. 1b). The ROC analysis did not show predictive roles for resistin and chemerin for T2DM. The AUC were 0.54 ± SE 0.05 (95% CI 0.44–0.63, *p* = 0.43) and 0.46 ± SE 0.05 (95% CI 0.37–0.56, *p* = 0.44) for resistin and chemerin respectively.

## Discussion

In this nested case–control study, among Asian Indians, we observed that higher baseline levels of serum visfatin and fetuin-A were associated with conversion of prediabetes to T2DM mediated by insulin resistance. These variables were strongly correlated and the conversion was significantly higher when their elevated levels coexisted.

The study cohort was chosen from a large prospective, randomised controlled trial that assessed the effectiveness of sustained lifestyle changes promoted by text messaging strategy in persons at high risk of developing diabetes. Middle - aged men and women with pre-existing metabolic abnormalities and impaired glucose regulation were selected. Of the two groups chosen for the analyses, individuals who converted to T2DM were markedly obese with upper body adiposity, higher body fat composition and raised fasting plasma glucose at baseline. Progressive, chronic-low grade inflammation could have been present in these persons.

In this cohort, baseline concentrations of serum visfatin were significantly raised among individuals with incident T2DM. Another South Asian cross-sectional study also showed raised visfatin levels in persons with newly diagnosed T2DM in comparison to normal individuals^20^. In these studies, serum visfatin was closely associated with obesity, in specific, abdominal obesity (increased WC), visceral fat mass and insulin resistance. A study from Egypt also showed increased serum visfatin concentrations in obese diabetes patients^21^. Positive correlations of visfatin with BMI, fasting insulin and HOMA-IR were reported. The parallel increase in circulating levels of visfatin and insulin could be due to a common association of these parameters with insulin resistance. A meta-analysis of 13 studies showed increased visfatin concentrations in participants with overt obesity, T2DM, metabolic syndrome, and cardiovascular diseases, which were positively associated with insulin resistance^22^. No gender difference in visfatin levels had been reported although gender differences in anthropometric parameters existed^23^.

It was interesting to note an association between serum visfatin with fetuin-A levels and their combined influence on the development of T2DM. Increased levels of FFAs which cause insulin resistance may be a link between the association of visfatin and fetuin-A in prediabetes.

We observed that fetuin-A was positively correlated with fasting glucose and insulin levels, insulin resistance and increased WC. No correlation was observed with BMI, body fat composition and lipid parameters. Elevated baseline concentrations of serum fetuin-A was independently associated with the risk of T2DM. Another prospective study among Asian Indians with prediabetes followed up for 12 months also had similar observations^24^. The study indicated that individuals progressing to diabetes had higher baseline fetuin-A concentrations in the presence of increased blood glucose values, pro-inflammatory markers, fatty liver index scores and non-alcoholic fatty liver disease in comparison to the non-progressors. A large prospective study among women in Unites States^25^ reported a 27% increased risk of T2DM per 100 µg/ml increment of plasma fetuin-A levels, independently of the influence of liver enzymes and other risk factors for diabetes. A recent meta-analysis of 7 studies from multi ethnic populations also showed positive association between fetuin-A levels and the risk of T2DM. It was concluded that one standard deviation increment of fetuin-A level was associated with a 23% greater risk relative (1.23 [95%CI 1.16–1.31], *p* < 0.001) of incident T2DM^26^.

Another recent study conducted among Chinese T2DM patients and their matched controls reported higher fetuin-A levels among obese T2DM compared to non-obese patients and normal subjects (*p* < 0.0001)^27^. Fetuin-A was found to correlate significantly with all metabolic parameters in obese normal and T2DM patients but not in non-obese patients. These results corroborate the link between fetuin-A and T2DM mediated by obesity and insulin resistance. However, our study also showed that serum Fetuin-A with its independent strong association with T2DM can be a potential predictor of incident T2DM in persons at risk.

Gender difference was not observed in levels of Fetuin-A^28^. Only one study reported that association of Fetuin-A with diabetes was stronger in women (Hazard Ratio, (HR) = 2.61, [95%CI 1.59–4.26] than among men (HR: 1.32, 95%CI 0.84–2.08)^29^. The findings discussed here were consistent with the early landmark EPIC Potsdam study that showed circulating fetuin-A levels to be predictive of incident T2DM through mechanisms related to insulin resistance and impaired β-cell function^30^.

Resistin, though highly associated with insulin resistance in persons with central adiposity and increased body fat, did not show an independent association with incident T2DM in our study cohort. There may be racial differences in the role of resistin as shown by prospective long-term studies in Western populations^31^. Higher levels of resistin were reported in women than in men^31,32^. It may be that resistin levels become significantly increased in obese and older persons and this could probably explain the lack of association in our cohort^33^.

We found chemerin to be positively correlated with BMI and body fat components but it was not an independent risk factor for T2DM. A study in Turkey also showed no significant differences in chemerin levels among T2DM, prediabetes and in normal subjects^34^. Several studies showed association of chemerin with obesity, insulin resistance, other components of metabolic syndrome and inflammation^19,35–37^. Such an association was noted even among healthy individuals in a study conducted in Germany^38^. We included chemerin in this study to explore the possibility of its involvement in diabetogenesis, as cardiometabolic syndrome is common among persons with prediabetes and diabetes. Despite a high prevalence of abnormal anthropometric variables and lipid profile in many of the participants, we did not find a significant association of chemerin with incidence of T2DM.

In summary, our findings showed that visfatin and fetuin-A were associated with the conversion to T2DM probably mediated by insulin resistance. As early predictors of T2DM, estimating the circulating concentrations of these two peptides would be useful in identifying persons at high risk of the disease and could aid in early interventional measures.

## Material and methods

### Recruitment of participants in the primary study

**Figure 2:**
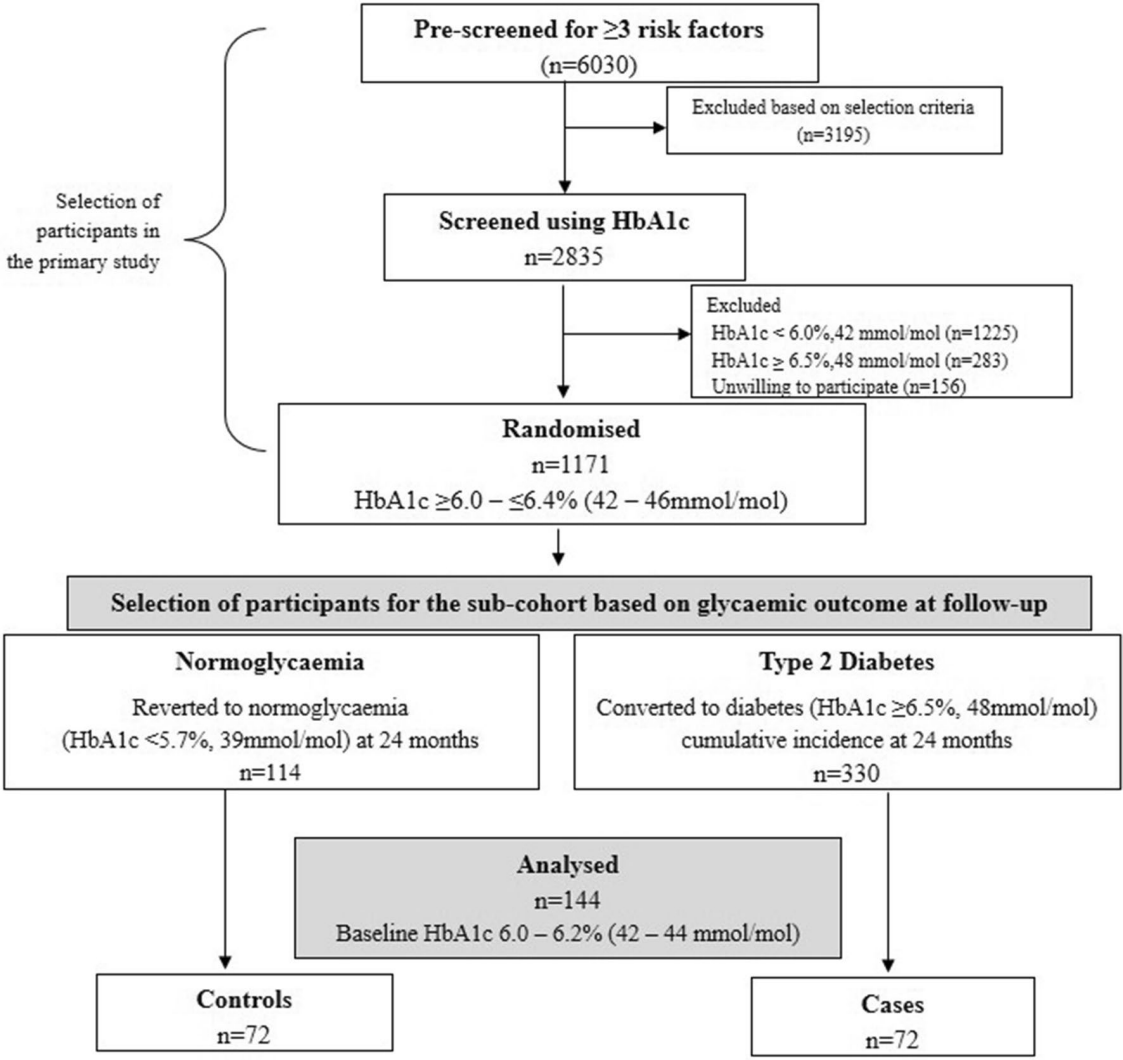
Steps involved in the selection of the study groups.

The primary study was undertaken in Asian Indian men and women aged 35–55 years in Chennai, Southern India, between April 2012 and November 2015^39^. A total of 6030 persons without a history of T2DM were pre-screened using non-invasive risk assessment to identify the high-risk group (Fig. 2). High risk was defined as the presence of ≥ 3 risk factors including: age ≥ 35 years, BMI ≥ 23 kg/m^2^, WC ≥ 90 cm in men and ≥ 80 cm in women, first degree family history of T2DM, history of hypertension or pre-diabetes and habitual sedentary behaviour. Participants in this category (n = 2835) underwent a test for hyperglycaemia using HbA1c measured by a point-of-care device (BioRad in2itTM system). Participants having values between ≥ 6.0% and ≤ 6.4% (≥ 42 to ≤ 46 mmol/mol) (n = 1171) were randomised in the study and followed up at 6 monthly intervals for 2 years^6,40^. At the end of 2 years, a total of 330 converted to T2DM (HbA1c ≥ 6.5% (48 mmol/mol)) and 114 reverted to normoglycaemia (HbA1c < 5.7% (39 mmol/mol)). An expert committee convened by the American Diabetes Association, the International Diabetes Federation and the European Association for the Study of Diabetes recommended the use of HbA1c for diagnosis of diabetes with a cut-off value of ≥ 6.5%,48 mmol/mol, a proposal ratified by the World Health Organization^41,42^. The participants provided a voluntary written informed consent at the start of the study.

### Sample size calculation

Sample size was calculated for each study parameter based on previously published studies^24,31,43,44^ comparing mean or median concentrations of each parameter with T2DM versus healthy controls. The expected difference from these calculations is demonstrated as significant at *p* < 0.05 and with 90% power. The calculated sample sizes suggested that a maximum of 50 participants in each group would suffice. We chose 72 samples each in cases and controls for this sub-analysis.

### Selection of samples for the sub-analysis

Selection of samples for the present analysis was based on the glycaemic outcome at the endpoint of the primary study. Persons with incidence of T2DM (HbA1c ≥ 6.5%, 48 mmol/mol) at 12 or 24 months visit were grouped as cases (n = 72) and those reverted to normoglycaemia, (HbA1c < 5.7% (39 mmol/mol) at 24 months were grouped as controls (n = 72). We chose samples with baseline HbA1c values between 6.0% (42 mmol/mol) and 6.2% (44 mmol/mol) since higher HbA1c values could be a confounder of conversion to T2DM. Samples (n = 144) were selected regardless of the randomized group in the primary study. Only HbA1c levels were used as the diagnostic criteria at all points of the study.

### Analytical methods

During the baseline and at review visits, anthropometry (BMI, WC) and HbA1c (in venous EDTA blood, using immunoturbidimetry) were measured. Lipids and FPG were measured at baseline and at annual visits. Biochemical analyses were done using an automated analyser (COBAS-6000, Roche system) with appropriate quality control measures. HOMA-IR was calculated using the formula: ((fasting insulin(mU/L) * fasting glucose(mmol/L))/22.5) as an index of insulin sensitivity and HOMA-β was calculated using the formula: ((20*fasting insulin(mU/L))/fasting glucose(mmol/L)-3.5) as an index of the β-cell secretion^45^. Fasting insulin was measured using chemiluminescence.

Body fat composition was assessed using the impedance meter (Tanita TBF-611 Body Fat Monitor; Syscon Instruments, India). Fasting blood samples were collected at baseline, 12 and 24 months, serum was separated and aliquots were stored in duplicates at − 80 °C for future estimations.

### Immunological assays

Circulating concentrations of visfatin (RayBiotech Inc, Norcross USA), resistin (Assay Pro LLC, USA), fetuin-A (Assay Pro LLC, USA) and chemerin (Quantikine ELISA, R& D Systems Inc.USA were estimated using commercially available enzyme linked immunosorbent assay (ELISAs) kits using freshly thawed serum samples.

### Ethical approval and study registration

The study was approved by the ethics committee of India Diabetes Research Foundation and Dr.A.Ramachandran’s Diabetes Hospitals, Chennai. All methods were carried out in accordance with the national guidelines of the Indian Council of Medical Research. The primary prevention trial was registered in www.ClinicalTrials.gov, NCT01570946 (03/03/2012) and the present study in the Clinical Trials Registry of India; CTRI/2018/03/012528 (13/03/2018).

### Statistical methods

Normally distributed variables were expressed as mean and ± SD. Median values and interquartile range (IQR) were reported for skewed variables. Intra and intergroup variations were tested using paired or unpaired t tests as relevant. For non-normally distributed variables, intra and intergroup comparisons were done using Wilcoxon’s signed rank test and Mann–Whitney U test respectively. Skewed variables were log transformed before analyses. Pearson correlation was performed to evaluate the association of visfatin, resistin, chemerin and fetuin-A with the probable risk variables. Multiple logistic regression analyses with stepwise addition were done to identify variables that were significantly associated with incidence of diabetes. The dependent variable was T2DM versus normoglycaemia. The variables that correlated with each study parameter were included in the respective regression equation as independent variables. The ROC analysis was done to assess the sensitivity and specificity of each study parameter to predict incident diabetes. Diagnostic accuracy was assessed by the area AUC. All analyses were performed with SPSS version 21.0.
